# Systematic comparison of germline variant calling pipelines cross multiple next-generation sequencers

**DOI:** 10.1038/s41598-019-45835-3

**Published:** 2019-06-27

**Authors:** Jiayun Chen, Xingsong Li, Hongbin Zhong, Yuhuan Meng, Hongli Du

**Affiliations:** School of Biology and Biological Engineering & Department of Biomedical Engineering, South China University of Technology, Guangzhou, China

**Keywords:** Next-generation sequencing, DNA sequencing

## Abstract

The development and innovation of next generation sequencing (NGS) and the subsequent analysis tools have gain popularity in scientific researches and clinical diagnostic applications. Hence, a systematic comparison of the sequencing platforms and variant calling pipelines could provide significant guidance to NGS-based scientific and clinical genomics. In this study, we compared the performance, concordance and operating efficiency of 27 combinations of sequencing platforms and variant calling pipelines, testing three variant calling pipelines—Genome Analysis Tool Kit HaplotypeCaller, Strelka2 and Samtools-Varscan2 for nine data sets for the NA12878 genome sequenced by different platforms including BGISEQ500, MGISEQ2000, HiSeq4000, NovaSeq and HiSeq Xten. For the variants calling performance of 12 combinations in WES datasets, all combinations displayed good performance in calling SNPs, with their F-scores entirely higher than 0.96, and their performance in calling INDELs varies from 0.75 to 0.91. And all 15 combinations in WGS datasets also manifested good performance, with F-scores in calling SNPs were entirely higher than 0.975 and their performance in calling INDELs varies from 0.71 to 0.93. All of these combinations manifested high concordance in variant identification, while the divergence of variants identification in WGS datasets were larger than that in WES datasets. We also down-sampled the original WES and WGS datasets at a series of gradient coverage across multiple platforms, then the variants calling period consumed by the three pipelines at each coverage were counted, respectively. For the GIAB datasets on both BGI and Illumina platforms, Strelka2 manifested its ultra-performance in detecting accuracy and processing efficiency compared with other two pipelines on each sequencing platform, which was recommended in the further promotion and application of next generation sequencing technology. The results of our researches will provide useful and comprehensive guidelines for personal or organizational researchers in reliable and consistent variants identification.

## Introduction

Revolutionary next generation sequencing (NGS) technologies have remarkably decreased the cost of genome sequencing and enabled the promotion of technology, leading to the brilliant achievements in genome sequencing projects such as 1000 genome project^1^ and HapMap project^2,3^. As the two main types of genome sequencing, whole-genome sequencing (WGS) and whole-exome sequencing (WES) have been widely implemented for the discovery of numerous genetic disease-associated genes and identification of drive mutations for specific types of cancer, which paved the way for the fundamental understanding how mutated genes affect disease phenotype and the further elucidation of pathogenic mechanism^4–6^. However, the current bottleneck of WGS and WES technology lies in the system’s data management methods and complex mutation call analysis rather than sequencing of the genome itself, which poses great challenges for genetic researchers. With the development of plentiful of tools, the performance on the specific part or the whole analysis process is continuously improved. A wealth of small genomic variants, including single-nucleotide polymorphisms (SNPs) and insertions and deletions (INDELs) are detected by various variants calling pipelines. Each of pipelines mainly consist of the quality assessment, read alignment, variant identification and annotation^7^, and different combinations of tools belonging to each part above-mentioned will result in the divergence of performance among pipelines and finally affect the interpretation of the variants calls. Therefore, the accurate identification of genomic variants and the standardization of benchmarking performance of different pipelines are critical for the genomics researches based on NGS technology.

A golden standard genotype dataset (sample NA12878) published by the Genome in a Bottle (GIAB) consortium is available for comparisons of variants calling pipelines^8^. Recently, several studies used the GIAB variant data for comparisons among different variants callers to choose the most appropriate pipeline for promotion and application^9–14^, while these analyses reported relative high divergence and substantially low concordance of called variants made by these pipelines, suggesting that there are several issues needed to be addressed. First, previous researches continuously initiated their research with a unitary sequencing (FASTQ file) or mapping (BAM file) data set, which made the data-specific effects difficult to exclude. In order to draw a conclusion that can be generalized on the genomes from various personal samples, evaluation of variants calling pipelines based on multiple datasets generated from various sequencing platforms systems is necessary. Second, these studies adopted measurement of Precision and Recall, or simply True-Positive (TP) and False-Positive (FP) to benchmark performance of different pipelines, which were not adequate to reflect the intrinsic trade-off between Precision and Recall and thus the more informative measurements such as F-score or the area under a precision-recall curve (APR) were needed. Third, both of the rapidly dropping cost of sequencing and the prevalent application of NGS analysis are continuously bringing about the production of large-scale datasets, which have gained researchers’ increasing attention to the unmet requirements on more efficient analysis pipelines and more powerful computational resources^15^. Therefore, besides precisely detecting the mutation site, evaluating the efficiency of the various detection pipelines by measuring the time-consuming is also a necessary part of systematic comparison among pipelines.

In this study, we obtained both WES and WGS datasets of the NA12878 cell-line sample generated from multiple sequencing platforms including HiSeq4000, NovaSeq, Xten from Illumina and BGISEQ500, MGISEQ2000 from BGI. After the quality control process by fastp^16^ and read alignment by Burrows-Wheeler Aligner (BWA), then we separately matched these analysis-ready datasets to three variants calling pipelines, including Genome Analysis Tool kit HaplotypeCaller (GATK-HC)^17,18^, Strelka2^19^ and Samtools-Varscan2^20,21^, to produce multiple “sequencer-pipeline” combinations. The NA12878 benchmark variant set^22^ was used to compare performance, concordance of each combination reflecting diverse sequencing platforms and variants calling pipelines, and generally these results should be more reliably applicable to personal genomics data for clinical diagnosis and other applications. Moreover, after down-sampling the original WES and WGS datasets at a series of gradient coverage, the evaluations of variant calling period of three pipelines in different combinations were also conducted.

We aimed to evaluate the accuracy and efficiency of the combinations of different sequencer and pipeline for small variants systematically, to identify the most precise and efficient combinations and define the optimal variants calling pipeline for each sequencing platform according to its performance, concordance, time-consuming, and to provide useful guidelines of reliable variants identification for personal or organizational researchers in genomes sequencing.

## Results

### Multiple sequencing datasets summary

**Figure 1:**
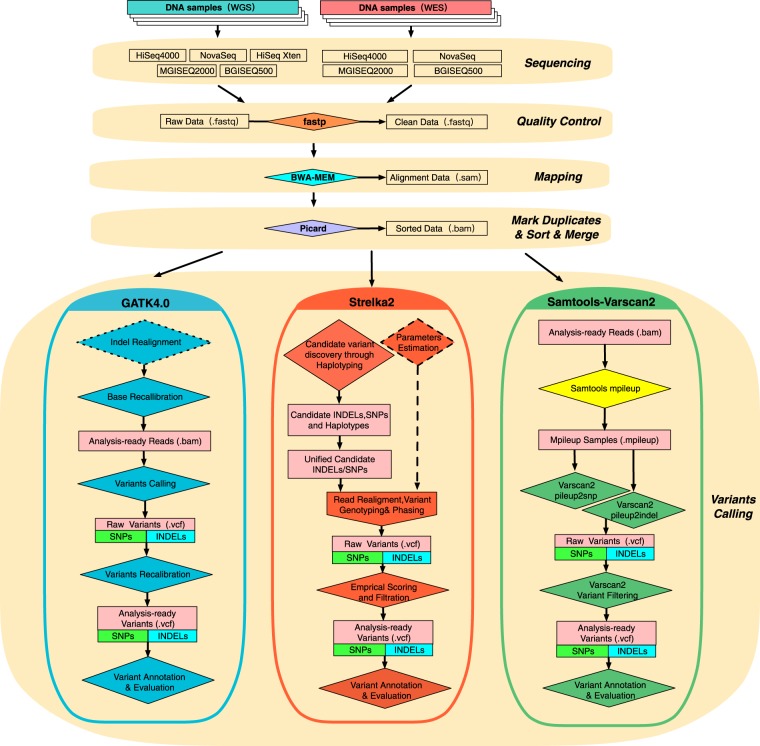
The flowchart of combinations using different sequencers and variant calling pipelines for germline variants. This workflow diagram reflects the designed comparison processes of the variants calling combinations. Key process for NGS data analysis were shown on the right. Squares in the flowchart represent data files, and rhombus indicate processes (the rhombus with dotted line mean that process were optional). After library preparation, samples are sequenced on multiple platforms to produce the raw datasets. The next steps are quality assessment and read alignment against a reference genome, followed by marking duplicates and sorting. Analysis-ready files of different platforms are analyzed by three variants calling pipelines using author-recommended parameters to generate VCF files, which were used for the final performance comparison of different combinations.

Applying the analysis process summarized in Fig. 1, we obtained NA12878 sequence datasets generated from multiple platforms including HiSeq4000, NovaSeq, HiSeq Xten from Illumina and BGISEQ500, MGISEQ2000 from BGI. There are 4 WES datasets obtained from HiSeq4000, NovaSeq, BGISEQ500 and MGI2SEQ000 and 5 WGS datasets from aforesaid sequencers plus HiSeq Xten. Considering that the low-quality reads (reads that had more than 10% bases with low-quality lower than 10) would affect the subsequently variants calling results, the raw FASTQ files of each datasets for WES and WGS were conducted filtering and trimming by fastp with default parameter settings.

In this way, we identified and trimmed 0.25%, 0.41%, 2.25% and 4.46% low-quality reads in BGISEQ500, NovaSeq and HiSeq4000 WES datasets, respectively. Similarly, 0.21%, 1.28%, 1.76%, 7.29% and 8.25% low-quality reads generated from MGISEQ2000, NovaSeq, BGISEQ500, HiSeq Xten and HiSeq4000 WGS datasets were separately cleaned out after quality control. Excluding these low-quality reads, we then analyzed the bases quality of these datasets. For all WES datasets, the percentages of bases quality for Q20 and Q30 were entirely higher than 95% and 89%, respectively. And for all WGS datasets, the percentages of bases quality for Q20 and Q30 were wholly higher than 92% and 83%, respectively (Tables 1 and S1).

**Table 1:** Summary of the multiple sequencing datasets in this study.

Sequencing Samples	Bases (Gbp)	Read (×10^6^)	Clean rare	>Q20	>Q30	GC content	Mean coverage
BGISEQ500-WES	29.41	294.30	0.41%	96.72%	89.14%	49.75%	328.49X
MGISEQ2000-WES	16.34	163.55	0.25%	98.18%	92.08%	49.71%	129.40X
HiSeq4000-WES	41.93	283.70	4.46%	97.36%	93.01%	50.63%	395.17X
NovaSeq-WES	25.88	178.87	2.25%	95.33%	92.67%	49.73%	241.52X
BGISEQ500-WGS	126.86	1270.02	1.76%	93.73%	83.33%	41.76%	41.03X
MGISEQ2000-WGS	137.36	1374.87	0.21%	96.17%	88.19%	41.76%	45.13X
HiSeq4000-WGS	191.00	1276.10	8.25%	95.90%	90.11%	41.69%	58.00X
NovaSeq-WGS	98.30	657.45	1.28%	95.89%	93.86%	41.61%	28.96X
HiSeq Xten-WGS	134.00	894.58	7.29%	94.50%	87.63%	40.71%	38.93X

The sequencing datasets was generated from multiple sequencers in both BGI and Illumina platforms. The length of reads in each of BGI and Illumina platforms were PE100 and PE150, respectively. The clean data rare indicated that the percentage of reads with low quality trimmed by fastp. “>Q20/Q30 percentage” indicates the percent of bases with quality score (−10*lg (error rate)) higher than 20 and 30 (indicating error rates of 1% and 1‰, respectively). The Mean sequence Depth indicates the average sequencing depth of datasets.

After quality control and assessment, 4 datasets for WES and 5 for WGS were subjected to further reads alignment and removing duplicates. For BGI platforms, the average read depth in BGISEQ500 platforms were 328.4X in WES dataset and 41.03X in WGS dataset, and in MGISEQ2000 platforms were 129.40X in WES dataset and 45.13X in WGS dataset, respectively. For Illumina platforms, the average read depth in HiSeq4000 were 395.17X and 58.00X in WES and WGS dataset, respectively. As for NovaSeq, the read depth of WES dataset was 241.52X and the WGS dataset was relative low (28.96X) while its coverage (>20X) was reached 92.1%. The average read depth of WGS in Xten platform was 38.93X (Tables 1 and S1). As the previous research recommended^23^, we reckoned that these datasets were available for the following analysis. All alignment data files above were performed variants identification including three variant calling pipelines: GATK4-HC (GATK), Strelka2(SK2) and Samtools-Varscan2(SV), which finally produce 27 Variants calling format (VCF) files in total for evaluation and comparison.

### Variants calling performance comparison of combinations in WES datasets

**Figure 2:**
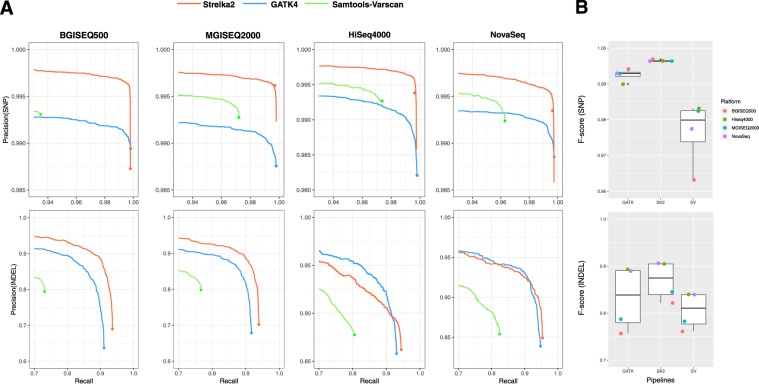
The summary of variants calling performances of multiple combinations in WES datasets. (**A**) Accuracy of SNPs (up) and INDELs (down) calls for BGISEQ500, MGISEQ2000, HiSeq4000, NovaSeq WES datasets. Large solid circles indicate the pass threshold of each combination. (**B**) Box plot indicates the distribution of F-score of multiple combinations in calling SNPs (up) and INDELs (down).

To assess the overall germline variants calling performance in WES datasets, we compared the germline-variant call accuracy of SK2, GATK and SV pipelines across 4 sequencing platforms. We found that SK2 manifested substantially higher precision than both other pipelines at almost all recall thresholds (>0.92) on overall WES datasets (Fig. 2A). Notably, as depicted in the precision-recall curve plot of SNPs, although the SV’s precision was higher than that of GATK at the recalled region, its constantly weaker recall was making the comprehensive performance inferior to that of GATK.

We also compared the distributions of F-scores of each combination on SNPs and INDELs, which were more informative than mere precision or sensitivity (Fig. 2B). For each sequencing platform, we found that the performance of the combinations with SK2 was better than that incorporated with GATK, then the group with GATK was superior to the one with SV, either in BGI platforms or Illumina platforms. And we also found that the deviation of F-scores in combinations incorporated with SK2 were smaller than other two counterparts, which means that the superior platform stability of SK2.

For the 12 combinations in calling SNPs, their F-scores in calling SNPs were entirely higher than 0.96. For BGI platforms, the BGISEQ500-SK2’s accuracy was considerably higher than that of BGI500-GATK and BGI500-SV, improving on the SNP F-score by 0.27% and 3.50%, respectively. And MGISEQ2000-SK2’s F score were also higher 0.35% and 1.42% than that of MGISEQ2000-GATK and MGI2000-SV combination, too. For Illumina platforms, the performance of incorporation with Strelka2 was still outperformed other two variants calling pipelines, with 0.19% higher F-score than that of GATK and 1.78% of SV in NovaSeq, and 0.52% of GATK and 1.22% of SV in HiSeq4000, respectively (Table S2).

Compared with SNPs calling, the performance of each pipeline in INDELs calling manifested larger divergence, whose F-scores were ranged from 0.75 to 0.91. Notably, all of BGI platforms were inferior to Illumina platforms in INDELs identification, which could be explained by their relative short length of reads (Pair-end 100 bp) compared with Pair-end 150 bp sequencing of Illumina platforms. For the identical sequencing platforms, the combinations incorporated with Strelka2 were outperformed other two variants calling pipelines as before, which showed substantially higher precision than other variant callers, with F-score exceed 1.29–8.50% compared with GATK and with SV (Table S2). Notably, affected by its relatively lower recall values, HiSeq4000-SV gained its middle position even it displayed the highest precision (=0.87) among all datasets. Similarly, the combination of BGI500-GATK achieved the lowest F-score due to its poor recall values, which means that parts of True-Positive variants were beyond the scope of identification.

### Variants calling concordance of multiple combinations in WES datasets

**Figure 3:**
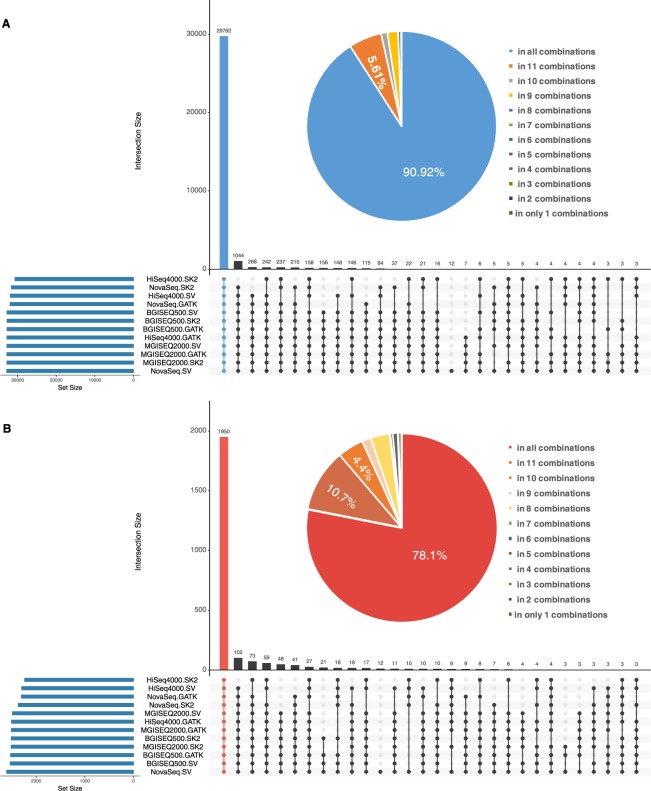
Intersection of variant Calling results of all combinations in SNPs and INDELs of WES datasets. The top bar-plot indicates the intersection size. This plot provides the number of variants that are uniquely called by one tool (a single point) or the numbers of variants called by many tools (two or more points). The bottom left plot indicates the set size. The linked points below display the intersecting sets of interest or which tools called variants. (**A**) UpSetR plot indicates intersection of variant calling results of all combinations in SNPs. (**B**) UpSetR plot indicates intersection of variant calling results of all combinations in INDELs.

The variant calling results of all 12 combinations in SNPs and INDELs were tested with the standard WES variants datasets based on the locus coordinates of each variant. If a variant was jointly detected by two or more combinations, this variant was ascribe to the intersection of these results of combinations. The distribution of the variants represent the concordance of different combinations. Upset Venn diagram in Fig. 3 depicted the intersection of the detected variants in the 12 combinations in SNPs and INDELs.

For the all combinations in SNPs, the NovaSeq-SV combination identify the largest size of SNPs variants while the combination of HiSeq4000-SK2 identify the lowest number of SNPs. All of 12 combinations jointly identified 29762 SNPs, which was almost 90.52% of standard WES SNPs’ amount. And SNPs called by 11 combinations and 10 combinations were 1836 (5.61%) and 351 (1.0%) in total, respectively (Fig. 3A). Therefore, over 97.13% of SNPs in WES dataset were to detected by at least 10 combinations. Notably, 12 SNPs variants were only called by the combination of NovaSeq-SV. The relative high overlapping variants set and the relative low solitary variants set revealed that larger agreement of these combinations in identified SNPs.

For the all combinations in INDELs, Still, the NovaSeq-SV combination identify the largest size of INDELs variants and the combination of HiSeq4000-SK2 identify the lowest number of INDELs while the order of set size differed with that in SNPs calling. 1950 identified INDELs were overlapping between all combinations, which was almost the 78.1% of standard WES INDELs’ amount. And INDELs called by 11 combinations and 10 combinations were 258 (10.7%) and 110 (4.4%) in total, respectively. At least 10 combinations were able to identify 93.2% INDELs in WES dataset. Similarly, there were 12 INDELs only detected by NovaSeq-SV, which revealed peculiarity of this combination in detecting variants. Even displaying larger divergence of concordance among combination compared with SNPs identification, the amount of variants detected by multiple combinations still indicates the relative high concordance of different combinations in INDELs calling (Fig. 3B). In summary, multiple combinations show widely agreement regarding the identification of SNPs and INDELs of WES datasets.

### Germline variants calling efficiency of multiple pipelines in WES datasets

**Figure 4:**
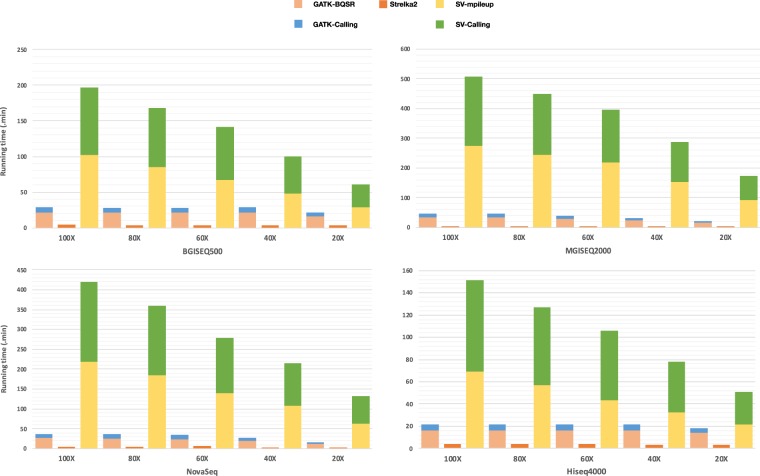
Variant calling runtime of multiple combinations in WES datasets. The variant calling runtime of each combination run on a Tianhe-2 supercomputer with 24 virtual CPUs and 88 GiB of memory. All combinations were configured to schedule tasks over all 24 virtual CPUs. The coverages of the down-sampled datasets were approximately 20X, 40X, 60X, 80X, 100X, respectively. Among them, variants calling in SK2 sets 24 threads, GATK used the default setting thread in BQSR and 24 threads setting in variants calling, and SV used the default setting thread in mpileup and variants calling.

To assess the operating efficiency of pipelines, we measured the whole variants calling period of each pipeline across multi-platforms. Considering that the WES datasets were generated from different platforms and there are certain operations overhead for variant calling on different bam files, thus we first down-sampled all the original WES datasets (the sorted and mark-duplicated BAM file) to the same coverage (100X). Then we again down-sampled the coverage of datasets to 20X (0.2 fold), 40X (0.4 fold), 60X (0.6 fold), 80X (0.8 fold) from the 100X datasets, respectively. After achieving WES datasets at a series of gradient coverage across multiple platforms, the time consumed by the three variants calling pipelines at each sequencing depth were counted separately (Fig. 4).

We benchmarked the running time of SK2 against GATK and SV. For BGI platforms, the runtime of the combinations with SK2 were higher 8.85 times than that with GATK and 67.16 times than SV on average. While for Illumina platform, the efficiencies of combination with SK2 were 6.63 times and 47.89 times higher than the combination with GATK and the combination with SV on average, respectively (Table S3).

### Variants calling performance comparison of combinations in WGS datasets

**Figure 5:**
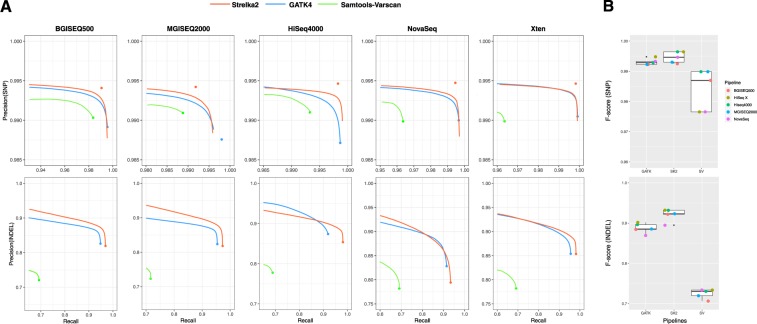
The summary of variants calling performances of multiple combinations in WGS datasets. (**A**) Accuracy of SNPs (up) and INDELs (down) calls for BGISEQ500, MGISEQ2000, HiSeq4000, NovaSeq and Xten WGS datasets. Large solid circles indicate the pass threshold of each combination. (**B**) Box plot indicates the distribution of F-score of multiple combinations in calling SNPs (up) and INDELs (down).

Still, we found that SK2 manifested substantially higher precision than both other two pipelines at almost all recall thresholds on overall WGS datasets. The overall germline variants calling performance in WGS datasets among 15 combinations were similar to that in WES datasets. For each sequencing platform, whether SNPs calling or INDEL calling, the combination incorporated with SK2 was still superior to other two variants calling groups (Fig. 5A). And we found that the deviations of F-scores in calling SNPs were larger than INDELs in each pipeline, and GATK and SK2 displayed better platform stability, with the lower deviation of F-scores compared with SV in calling SNPs and INDELs, respectively (Fig. 5B).

For SNP variant calls, all these combinations manifested good performance, with their F-scores in calling SNPs were entirely higher than 0.975. Both Xten-SK2 and HiSeq4000-SK2 showed the highest F-score (0.996). Both of BGI platforms and Illumina platforms, the group combined with SK2 was superior to the group incorporated with GATK in SNPs calling, then the combination with GATK was surpassed the one with SV. Notably, we founded that a significant drop in F-sore of the Xten-SV and NovaSeq-SV combinations (2.04% and 1.85%, respectively) compared with their SK2 counterparts. For each sequencer in BGI platforms, the margin of performance between any two combination of pipelines were smaller than that of any sequencer in Illumina platforms, which demonstrated that choosing different pipeline will cause greater impact to Illumina platforms.

For INDEL variant calls, both GATK and SK2 displayed better performance, with their F-scores in calling INDELs were entirely higher than 0.85. Thereinto, the combinations of Xten-SK2 and HiSeq4000-SK2 manifested the highest F-score (0.931) in consonance with that in SNP calling. For Illumina platforms, the performance of incorporation with Strelka2 was still outperformed other two variants calling pipelines, with 2.93–9.67% higher F-score than that of GATK and 21.96–34.79% of SV. And similar trend exists in BGI platforms, the F-score of combinations with SK2 were 4.26% higher than that of GATK and 28.27–30.52% of SV. Notably, we found that even have longer length of reads (PE150bp), the combinations of NovaSeq-GATK and NovaSeq-SK2 displayed the lower F-score compared with other combinations (Table S4).

In conclusion, we found that the order of performance that the combinations with SK2 were better than that incorporated with GATK, then the group with GATK was superior to the one with SV, either in BGI platforms or Illumina platforms. This results raveled that Strelka2 was not only considered to be the optimal variant calling pipeline in WES datasets, but applicable to the WGS datasets. Moreover, the distinguished performance in both SNP (F-score = 0.996) and INDEL (F-score = 0.931) made the combination of Xten-SK2 and HiSeq4000-SK2 optimal for calling variants of WGS datasets.

### Variants calling concordance of multiple combinations in WGS datasets

**Figure 6:**
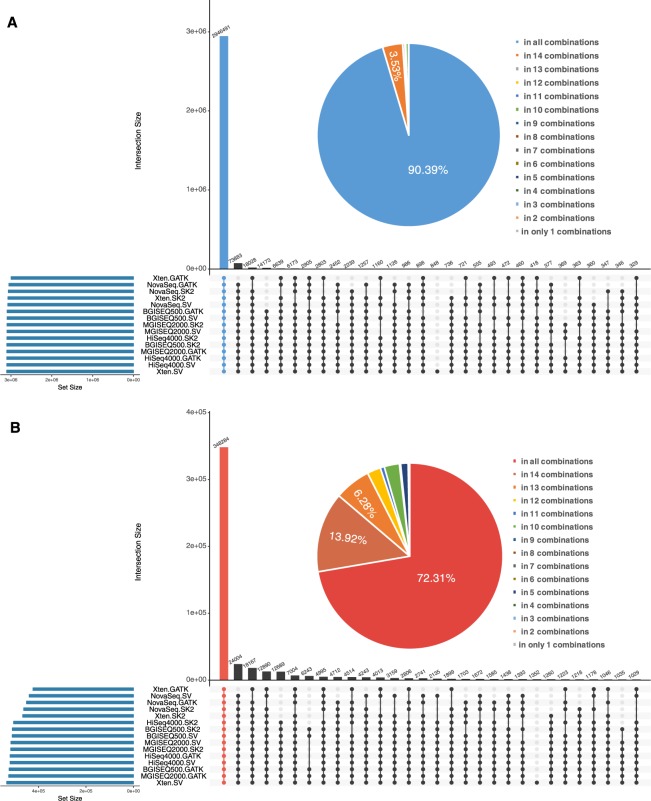
Intersection of variant Calling Results of all Combinations in SNPs and INDELs of WGS datasets. The top bar-plot indicates the intersection size. This plot provides the number of variants that are uniquely called by one tool (a single point) or the numbers of variants called by many tools (two or more points). The bottom left plot indicates the set size. The linked points below display the intersecting sets of interest or which tools called variants. (**A**) UpSetR plot indicates intersection of variant calling results of all combinations in SNPs. (**B**) UpSetR plot indicates intersection of variant calling results of all combinations in INDELs.

As with WES datasets, the variant calling results of all combinations in SNPs and INDELs of WGS datasets were compared with the standard WGS variants datasets based on the locus coordinates of each variant. Upset Venn diagram demonstrated the intersection of the detected variants in the all combinations in SNPs (Fig. 6A) and INDELs (Fig. 6B).

For the all combinations in SNPs, the Xten-SV combination identified the largest size of SNPs variants while the combination of Xten-GATK identify the lowest number of SNPs. All of 15 combinations jointly identified 2946491 SNPs, which was almost 90.39% of standard WGS SNPs’ amount. And SNPs called by 14 combinations and 13 combinations were 108986 (3.53%) and 11658 (0.3%) in total, respectively. Therefore, over 94.22% of SNPs in WGS dataset were detected by at least 10 combinations. Notably, 848 SNPs were only called by the combination of Xten-SV. The relative high overlapping variants set and the relative low solitary variants set revealed that larger agreement of these combinations in identified SNPs.

For the all combinations in INDELs, Still, the Xten-SV combination identified the largest size of INDELs variants and the combination of Xten-GATK identified the lowest number of INDELs while the order of set size differed with that in INDELs calling. 348284 identified INDELs were overlapping between all combinations, which was almost the 72.31% of standard WGS INDELs’ amount. And INDELs called by 14 combinations and 13 combinations were 67060 (13.92%) and 30264 (4.4%) in total, respectively. At least 13 combinations were able to identify 90.63% INDELs in WGS dataset. Similarly, there were 15 INDELs only detected by Xten-SV, and we found the largest size of combinations were inclined to be peculiar in detecting variants. As in the WES datasets, there are larger divergences of concordance in INDELs identification among combination compared with SNPs identification. The amount of variants detected by multiple combinations still indicates the relative high concordance of different combinations in INDELs calling. In summary, multiple combinations also show widely agreement regarding the identification of SNPs and INDELs of WGS datasets.

### Germline variants calling efficiency of multiple pipelines in WGS datasets

**Figure 7:**
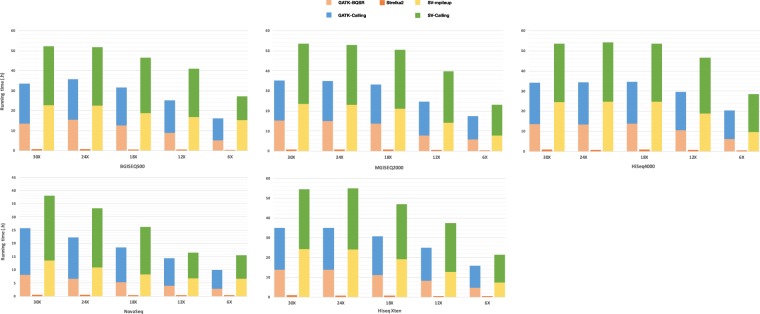
Variant calling runtime of multiple combinations in WGS datasets. The storage footprints and variant calling runtime of each combination run on a Tianhe-2 supercomputer with 24 virtual CPUs and 88 GiB of memory. All combinations were configured to schedule tasks over all 24 virtual CPUs. The coverages of the down-sampled datasets were approximately 6X, 12X, 18X, 24X, 30X, respectively. Among them, variants calling in SK2 sets 24 threads, GATK used the default setting thread in BQSR and 24 threads setting in variants calling, and SV used the default setting thread in mpileup and variants calling.

To assess the operating efficiency of pipelines in WGS datasets, we also measured the whole running time of each pipeline across multi-platforms. And the same thing goes for the WGS datasets, we first down-sampled all the original WGS datasets (the sorted and mark-duplicated BAM file) to the same coverage (30X). Then we again down-sampled the coverage of datasets to 6X (0.2 fold), 12X (0.4 fold), 18X (0.6 fold), 24X (0.8 fold) from the 30X datasets, respectively. After achieving WGS datasets at a series of gradient coverage across multiple platforms, the running time of the three variants calling pipelines at each sequencing depth were counted separately (Fig. 7).

As with WES datasets, we benchmarked the running time of SK2 against GATK and SV. For BGI platforms, the runtime of the combinations with SK2 were higher 45.52 times than that with GATK and 69.49 times than SV on average. While for Illumina platform, the efficiencies of combination with SK2 were 41.94 times and 62.49 times higher than the combination with GATK and the combination with SV on average, respectively (Table S5).

## Discussion

In the past several years, with the development of NGS sequencers such Illumina and BGI^24,25^, the application of WES or WGS to identify variants of genome became accessible for pubic and individuals. To practice variants identification, various variants calling tools have been developed and constituted different pipelines, and several research about performance comparison of pipelines have been conducted to select the best pipeline. However, the data-specific effects and the lack of systematic metrics bring these studies some limitations, and a systematic performance comparison among variants calling method for each platform is necessary. Therefore, we performed a systematic performance comparison of pipelines across multiple sequencers by using the benchmark variant calls^22^.

In this study, after obtaining one individual (NA12878) from the 1000 genome project in parallel with the BGI500, MGISEQ2000, HiSeq4000, NovaSeq and Xten platforms (the former four samples had both WES and WGS datasets, the last one only had WGS datasets), we measure the base quality (Q20&Q30) and the average sequencing depth of each datasets, even their quality and sequencing depth varies due to their difference in sequencing performance and experimental operations, all these dataset were meet the requirements for the further analysis^26^. Using a set of reliable sequence variants for sample NA12878 released by GIAB for benchmarking performance in calling variants, we compared three popular pipelines (GATK-HC, Strelka2 and Samtools-Varscan2) across 4 WES datasets and 5 WGS datasets aforesaid to form 27 combinations, which totally produced 27 VCF files for further evaluation and comparison.

For the variants calling performance of 12 combinations in WES datasets, all combinations displayed good performance in calling SNPs, with their F-scores were entirely higher than 0.96, and their performance in calling INDELs varies from 0.75 to 0.91. And all 15 combinations in WGS datasets also manifested good performance, with F-scores in calling SNPs were entirely higher than 0.975 and their performance in calling INDELs varies from 0.71 to 0.93. For WES datasets, the BGI platforms displayed the superior performance in SNPs calling while Illumina platforms manifested the better variants calling performance in INDELs calling, which could be explained by their divergence in sequencing strategy that producing different length of reads (all BGI platforms were 100 base pair read length while all Illumina platforms were 150 base pair read length). The read length effects, as a key factor between two platforms, would bring alignment bias and error which are higher for short reads and ultimately affect the variants calling especially the INDELs identification^27^. For WGS datasets, both Xten-SK2 and HiSeq4000-SK2 had the highest F-scores in SNP and INDELs calling across all combinations.

With the consideration that the sequencing depth of dataset would bring effect to the results^26^, we found that the mean sequencing depth of MGISEQ2000 was only 129.40X which is much lower than other three counterparts, while its performance of SNPs identification was get closer to BGISEQ500 (whose sequencing depth was 2.53 times than MGISEQ2000) and better than HiSeq and NovaSeq (3.06 and 1.86 times than MGISEQ2000, respectively). As for INDELs identification, the MGISEQ2000 outperformed than BGISEQ500 even their read length were all 100 base pair. The similar result happens at the NovaSeq dataset in WGS, which outperformed than BGI500 and MGISEQ2000 in SNPs identification even it has the lowest sequencing depth. Therefore, we assume that the sequencing depth beyond a certain range would emit little effects in promoting the performance in variant identification especially in SNPs calling, which is instructive in setting the sequencing depth and cost under reasonable ranges. And this viewpoint was needed to further test and validation to draw a more comprehensive conclusion.

For each sequencing platform, we found that the performance of the combinations with SK2 was almost better than that incorporated with GATK, then the group with GATK was superior to the one with SV, either in BGI platforms or Illumina platforms. The germline analysis of SK2 introduces haplotype-model to reduce the effects of sequencing noise, incorrect read mapping, and inconsistent alignment. A tiered haplotype-modeling approach were used to estimate both INDEL variant mutation rates and INDEL noise rates from a set of error processes, which empower its high accuracy and sensitivity in detecting variants. However, considering that the trained models of Strelka2 are trained on known variant features we could not exclude the possibility that GIAB datasets has bias effect toward Strelka2 in variants calls. Besides, whether Strelka2 is capable of identifying ‘real’ rare variation in unsequenced genomes datasets needs further validation. The conservative detection strategy of SV ensure high precision in SNPs identification, while the inferior sensitivity of INDELs detection is strongly impaired to this pipeline, making this pipeline the weakest performance on accurate detection of INDELs. Besides, considering that the golden standard variants were made based on GATK-UnifiedGenotyper, GATK-HC and Cortex, the possibility that the golden standard has bias effect toward GATK-HC in variants calls, which ultimately leads to some combinations incorporated with GATK were outperformed other combinations combined with two pipelines could not be excluded^8,11^.

We also analyzed the concordance of SNPs and INDELs identification in the three pipelines for each sequencing platforms of WES and WGS datasets by using Upset Venn plot^28^. In summary, multiple combinations show widely agreement regarding the identification of SNPs and INDELs. However, the divergences among INDELs identification was always larger than SNPs identification, which could be mainly explained by their different read length. As for solitary variants set, we found that 23 small variants were merely detected by NovaSeq-SV in WES datasets and 861 small variants were only identified by Xten-SV, and we found that the largest size of combinations were inclined to be peculiar in detecting variants, which could be deemed to the peculiar proportion of variants enlarged the set size of this combination. Notably, there are many combination miss in detecting some part of variants that were able to be detected by other combinations, which impair its recall (sensitivity), consequently due attention should be paid in interpreting results on a single data set^12^. Overall, all combinations show a relative high concordance to each other in their capacities to identify germline SNPs and INDELs^11,26^.

Considering that the runtime of pipelines also are vital to deploy a certain variants calling pipeline in such various analysis platforms, we also assessed the variants calling running time for down-sampled datasets at a series of gradient coverage across multiple platforms. Both WES and WGS datasets, we founded that the processing speed of the combinations with SK2 were much higher than that with GATK or SV on every coverage. Strelka2 applies read likelihoods and maximum alignment-specific likelihood to avoid the computational cost of a complete implementation, which brings it additional runtime improvement. Besides, Strelka2 is allegedly capable of jointly genotyping up to ten samples, and its runtime was approximately linear as a function of total sequencing depth across all input samples^19^. Therefore, Strelka2 performs with extremely high efficiency to process aligned reads in compressed BAM format to high quality variant calls in VCF format, which bring it great advantage in being deployed in various NGS platforms.

Taken together, for each sequencing platform, we currently recommended Strelka2 as the optimal analysis pipeline due to its ultra-performance in detecting accuracy, high concordance and fast processing speed. We conclude that our researches will provide useful and comprehensive guidelines of reliable and consistent variants identification for personal or organizational researchers in translating the variant information into genomic medicine.

## Methods and Materials

### Sample acquisition and library preparation

We ordered 50 μg NA12878 cell line genomic DNA (Cat No. GM12878, Coriell), and the concentration was detected by Qubit fluorometer 3.0 (Invitrogen), the integrity and purification was detected by 1% Agarose Gel Electrophoresis, ensured to be of high quality with no visible degradation. After sample testing, the genomic DNA was constructed as Illumina & BGISEQ exome library and whole genome sequencing library. For Illumina library construction, 1 μg NA12878 genomic DNA was fragmented by Covaris E220 to DNA fragments, between 100 bp and 500 bp (for exome library), or between 150 bp and 800 bp (for whole genome sequencing library), respectively, and the Illumina adapter was ligated to both ends of DNA fragments used SureSelectXT Reagent Kit (Cat No. G9611A, Agilent), PCR amplification were applied to each sample after ligation, then the whole genome sequencing library was finished. On the other hands, the exome library was captured using the Human All Exon V5 Target Enrichment Baits (Cat No. 519-6216, Agilent). For BGISEQ exome and whole genome sequencing library construction, 1 μg genomic DNA was fragmented by Covaris E220 to DNA fragments between 50 bp and 400 bp, and then the DNA fragments was selected to between 100 bp and 300 bp by AMPure XP beads (AGENCOURT). After that, we used the MGIEasy™ DNA Library Prep Kit V1 (Cat No. 85-05533-00, BGI) to construct BGISEQ library, then the whole genome sequencing library was finished. Meanwhile, the exome library was captured using the Human All Exon V5 Target Enrichment Baits (Cat No. 519-6216, Agilent). Finally, All BGISEQ library were circularised to generate single stranded DNA circles.

### Data sets from multiple sequencing platforms

All prepared library was sent to BGI for sequencing. The Illumina exome and whole genome sequencing library was sequenced for pair end 150 on the HiSeq Xten, HiSeq4000 and NovaSeq sequencing platform. In contrast the BGISEQ exome and whole genome sequencing was sequenced for pair-end 100 bp on the BGISEQ-500 and MGISEQ-2000 sequencing platform. To avoid biased results by NGS platform, we used a similar two number of data sets sequenced by HiSeq4000, NovaSeq, BGI500 and MGISEQ2000, respectively. However, there was only one NA12878 whole genome sequence data set for Xten available for this study. Nine sequenced reads data sets for NA12878 were available for downloading from public databases: Sequence Read Archive (SRA) (https://www.ncbi.nlm.nih.gov/bioproject/PRJNA511646/). Short reads of each data set for WES and WGS were conducted filtering and trimming by fastp^16^.

### Read alignment and pretreatment

After quality assessment, the sequence reads were mapped them to the GRCh38 reference genome through BWA-MEM (0.7.17), and then aligned reads were converted to bam files of the binary version of sequenced alignment/map files (known as SAM files) with Samtools (v1.2). Alignment was done with default parameter settings. For each data sets, we used MarkDuplicate to remove duplicates in each bam files, Sorting based on genome position and Merge if necessary in Picard (v2.18.11) to prepare the analysis-ready datasets.

### Identifying variants using three variant calling pipelines

We analyzed mutations of each aligned datasets by using three variant calling pipelines: (1) Genome Analysis Tool Kit- HaplotypeCaller (v4.0.8.1), (2) Strelka2 (v2.9.7) and (3) Samtools-Varscan2 (v1.2 and v2.3.9, respectively). The former two pipelines call SNPs and INDELs jointly while SV detects SNPs and INDELs separately. Among them, GATK and SV were run via the recommended parameter of each variant caller, and SK2 was run with the default settings. For GATK pipeline, This workflow was applied using best practices described by the GATK developers: https://software.broadinstitute.org/gatk/best-practices, We performed base quality score recalibration (BQSR) on the raw BAM files, which used the default parameters or suggested input files, such as the most recent dbSNP VCF file, HapMap genotype file and OMNI 2.5 genotype VCF file. And the raw variants identified by the genotyping tool were refined using VariantFiltration, with parameters QualByDepth, FisherStrand, StrandOddsRatio, RMSMappingQuality, MQRankSum and ReadPosRankSum were <2.0, <40.0, >60.0, >3.0, <12.5 and <−8.0 as recommended, respectively.

For calling variants by SK2, we used mark-duplicated BAM files, described in the previous section without any additional process. And SK2 was run with the default settings. For SV pipeline, the analysis-ready datasets from GATK were performed Samtools mpileup functionality and run with a filter on mapping quality. Only reads with mapping quality 1 or higher and the max depth below 30000 were included in the pileup information. SNPs and INDELs were called by two separate functions of Varscan2, namely: pileup2snp and pileup2indel. min-coverage Minimum was set to 8, minimum variant allele frequency was set to 0.2 and p-value threshold was set to 0.1. For other parameters default settings were applied. After running the three variant callers for each of data sets, we regularized the 27 different variant calls files, in which 12 for WES and 15 for WGS, to the same variant calling format (VCF) that represented the results of each combination of sequencers-pipelines so as to the conduct further performance evaluation and comparison. All methods were tested on Linux CentOS 7.

### Evaluation of performance in variant-calling accuracy

For benchmarking variant calls identified by multiple combinations, the highly confident variant calls including SNPs and INDELs provided by NIST GIAB consortium (http://www.genomeinabottle.org) were used as the gold standard variants set in this study. Results were compared with high-confidence calls from GIAB truth sets in hap.py (https://github.com/Illumina/hap.py). To assess the performance of variant calling pipelines, we used precision-recall curves. We defined true positive (TP), true negative (TN), false positive (FP), and false negative (FN) variants as follows:

1. TP: variants called by a variant caller in high confident regions as the same genotype as the gold standard data
2. TN: reference alleles in high confident regions other than gold standard variants
3. FP: variants called by a variant caller in high confident regions but not as the same genotype as the gold standard data
4. FN: gold standard variants in high confident that were not called by a variant caller
5. Precision: TP/(TP + FP)
6. Recall: TP/(TP + FN)
7. F-score: 2* Precision*Recall/(Precision + Recall)

### Datasets down-sampling and measurement of running time

We used DownsampleSam command in Picard (v2.18.11) to down-sample the analysis-ready datasets (the sorted and mark-duplicated BAM file). Strategy was set to default, accuracy was set to 0.0001, and probabilities vary according to the coverage of down-sampled datasets. We first down-sampled the original WES and WGS datasets to the same coverage (WES in 100X and WGS in 30X), Then again down-sample the coverage of datasets above to a series of gradient coverage (WES in 20, 40, 60, 80X and WGS in 6X, 12X, 18X, 24X) across multiple platforms, then the variants calling period consumed by the three pipelines at each coverage were counted, respectively.

For Strelka2, GATK4 (including BQSR step and calling step) and Samtools-Varscan2 (including mpileup step and calling step), the wallclock time was recorded by using Time command in shell script and parsed in Linux logfile. Note that the runtime of the entire variants calling workflow was higher due to additional steps other than variant calling, such as preparation for parallelization, quality control and variant annotation. We reported only the time elapsed during variant calling.

## Supplementary information

Supplement Materials

## Data Availability

The raw sequencing reads during the current study are available in the NCBI SRA database (https://www.ncbi.nlm.nih.gov/bioproject/PRJNA511646/).
