# Supplementary material for: Systematic comparison of germline variant calling pipelines cross multiple next-generation sequencers
Source: Sci Rep. 2019 Jun 27;9:9345. doi: 10.1038/s41598-019-45835-3 (PMC6597787; doi:10.1038/s41598-019-45835-3)
Supplement: Supplementary file 1 — Supplement Materials [file 41598_2019_45835_MOESM1_ESM.pdf]

Supplement Materials

Systematic comparison of germline variant calling pipelines cross multiple next-generation sequencers

Jiayun Chen[1], Xingsong Li[1], Hongbin Zhong[1], Yuhuan Meng[1*], Hongli Du[1*]

1. School of Biology and Biological Engineering & Department of Biomedical Engineering, South China University of Technology, Guangzhou, China

# Correspondence address should be addressed to Hongli Du (E-mail:hldu@scut.edu.cn） or Yuhuan Meng(E-mail: meng.yuhuan@mail.scut.edu.cn)

(1) Table.1 | Statistics results of samples sequencing and mapping analysis.

Sequencing Samples*	Raw Reads ($x10^6$)	Raw Bases (Gbp)	Clean Reads ($x10^6$)	Clean Bases (Gbp)	Clean data rare	> Q20 (Raw)	>Q30 (Raw)	> Q20 (Clean)	> Q30 (Clean)	GC Content (Clean)	Mean sequence Depth(X)	Coverage （>0X）	Coverage （>10X）	Coverage （>20X）	Coverage （>30X）	Coverage （>50X）	Coverage （>100X）	Coverage （>150X）
BGISEQ500-WES	300.92	30.09	294.3	29.41	0.41%	96.44%	88.82%	96.72%	89.14%	49.75%	328.49	100.0%	99.9%	——	99.4%	98.2%	91.3%	80.7%
MGISEQ2000-WES	164.94	16.49	163.55	16.34	0.25%	98.01%	91.89%	98.18%	92.08%	49.71%	129.40	100.0%	99.8%	——	97.5%	89.6%	55.8%	29.9%
HiSeq4000-WES	297.15	44.57	283.7	41.93	4.46%	95.97%	91.03%	97.36%	93.01%	50.63%	395.17	100.0%	99.7%	——	98.9%	97.4%	91.6%	85.6%
NovaSeq-WES	183.35	27.5	178.87	25.88	2.25%	94.50%	91.58%	95.33%	92.67%	49.73%	241.52	100.0%	99.7%	——	99.1%	97.7%	87.3%	71.6%
BGISEQ500-WGS	1292.87	129.28	1270.02	126.86	1.76%	93.17%	82.58%	93.73%	83.33%	41.76%	41.03	100.0%	100.0%	99.5%	91.4%	16.2%	——	——
MGISEQ2000-WGS	1381.54	138.15	1374.87	137.36	0.21%	96.09%	88.09%	96.17%	88.19%	41.76%	45.13	100.0%	100.0%	99.7%	96.4%	30.5%	——	——
HiSeq4000-WGS	1392.35	208.85	1276.1	191	8.25%	93.27%	86.61%	95.90%	90.11%	41.69%	58.00	100.0%	100.0%	99.9%	99.1%	77.4%	——	——
NovaSeq-WGS	666.69	100	657.45	98.3	1.28%	95.50%	93.38%	95.89%	93.86%	41.61%	28.96	100.0%	99.5%	92.1%	45.4%	0.5%	——	——
HiSeq X-WGS	962.39	144.36	894.58	134	7.29%	92.62%	84.88%	94.50%	87.63%	40.71%	38.93	100.0%	100.0%	99.7%	91.5%	6.8%	——	——

*The sequencing datasets was generated from multiple sequencers in both BGI and Illumina platforms. The length of reads in each of BGI and Illumina platforms were PE100 and PE150, respectively. The clean data rare indicated that the percentage of reads with low quality trimmed by fastp. ">Q20/Q30 percentage" indicates the percent of bases with quality score (-10*lg(error rate)) higher than 20 and 30(indicating error rates of 1% and 1‰, respectively). The Mean sequence Depth indicates the average sequencing depth of datasets. The coverage rate indicates the proportion or reads with exceeded depth mapping in the reference genome.

(2) Table.2 | Summary of variant calling performances by 12 combination in WES dataset

Variant Type	Metrics	BGISEQ500			MGISEQ2000			Hiseq4000			NovaSeq		
		GATK	SK2	SV	GATK	SK2	SV	GATK	SK2	SV	GATK	SK2	SV
SNPs	True Positive	32514	32475	30450	32497	32453	31663	32505	32453	31716	32487	32448	31360
	False Positive	56	95	2120	73	117	907	65	117	854	83	122	1210
	False Negative	326	111	212	398	121	231	593	202	234	377	213	241
	Precision	0.998281	0.997083	0.934909	0.997759	0.996408	0.972152	0.998004	0.996408	0.97378	0.997452	0.996254	0.962849
	Recall	0.990074	0.996595	0.993086	0.987902	0.996286	0.992757	0.982086	0.993816	0.992676	0.98853	0.99348	0.992374
	F-score	0.994161	0.996839	0.96312	0.992806	0.996347	0.982347	0.989981	0.99511	0.983137	0.992971	0.994865	0.977389
INDELs	True Positive	2306	2336	1833	2293	2315	1922	2337	2349	2020	2380	2379	2066
	False Positive	206	176	674	219	197	585	175	163	487	132	133	441
	False Negative	1285	844	475	1029	655	486	390	337	282	461	365	353
	Precision	0.917994	0.929936	0.731153	0.912818	0.921576	0.766653	0.930334	0.935111	0.805744	0.947452	0.947054	0.824093
	Recall	0.644241	0.735755	0.794194	0.691824	0.780422	0.798173	0.857923	0.875231	0.877498	0.838868	0.867849	0.854072
	F-score	0.757133	0.821527	0.761371	0.787103	0.845146	0.782096	0.892663	0.904181	0.840091	0.88986	0.905723	0.838814

We defined true positive (TP), true negative (TN), false positive (FP), and false negative (FN) variants as follows: (1)TP: variants called by a variant caller in high confident regions as the same genotype as the gold standard data; (2)TN: reference alleles in high confident regions other than gold standard variants; (3)FP: variants called by a variant caller in high confident regions but not as the same genotype as the gold standard data; (4)FN: gold standard variants in high confident that were not called by a variant caller; (5)Precision: TP/(TP+FP); (6)Recall: TP/(TP+FN); (7)F-score: 2* Precision*Recall/(Precision+Recall)

(2) Table.3 | Summary of running time of combinations in WES datasets.

Platforms	Coverage	GATK4		Strelka2	Samtools-Varscan	
		BQSR	Calling	Calling	Mpileup	Calling
BGISEQ500	100X	21m34.55s	6m48.451s	4m4.854s	101m50.462s	94m75.41s
	80X	21m21.20s	6m45.209s	3m19.792s	85m34.626s	82m40.67s
	60X	21m20.68s	6m47.953s	3m19.454s	66m59.790s	74m46.46s
	40X	21m35.84s	6m46.237s	3m49.919s	48m24.140s	51m49.24s
	20X	16m17.59s	5m2.217s	2m49.948s	28m54.902s	31m67.85s
MGISEQ2000	100X	33m19.42s	13m3.053s	4m34.395s	273m51.277s	233m49.68s
	80X	33m49.77s	12m38.147s	4m49.408s	245m14.109s	203m92.94s
	60X	28m72.89s	10m30.827s	3m34.978s	218m46.512s	176m59.30s
	40X	22m73.61s	8m10.509s	3m4.569s	154m9.382s	131m96.80s
	20X	14m74.00s	5m2.123s	2m19.620s	93m0.452s	79m73.70s
NovaSeq	100X	25m92.19s	10m49.583s	4m34.578s	218m51.660s	200m96.72s
	80X	25m70.27s	10m45.391s	4m19.417s	184m42.671s	174m58.41s
	60X	23m75.86s	9m49.527s	5m34.631s	139m16.746s	139m42.84s
	40X	18m57.95s	7m17.576s	3m19.596s	107m5.700s	107m78.43s
	20X	11m62.03s	4m37.398s	2m34.183s	63m24.312s	68m36.78s
HiSeq4000	100X	16m13.08s	5m22.914s	4m4.468s	69m21.966s	81m53.52s
	80X	16m22.29s	5m26.317s	3m50.210s	56m47.047s	69m63.97s
	60X	15m63.87s	5m23.070s	3m49.555s	43m26.071s	61m71.33s
	40X	15m67.15s	5m36.125s	3m19.663s	32m19.994s	45m60.00s
	20X	13m54.98s	4m37.865s	3m4.478s	21m23.222s	28m68.01s

The variant calling efficiency of each combination run on a Tianhe-2 supercomputer with 24 virtual CPUs and 88 GiB of memory. All combinations were configured to schedule tasks over all 24 virtual CPUs. The coverages of the down-sampled datasets were approximately 20X, 40X, 60X, 80X, 100X, respectively. Among them, variants calling in SK2 sets 24 threads, GATK used the default setting thread in BQSR and 24 threads setting in variants calling,

and SV used the default setting thread in mpileup and variants calling.

(4) Table.4 | Summary of variant calling performances by 15 combination in WGS datasets.

Variant Type	Metrics	BGISEQ500			MGISEQ2000			Hiseq4000			NovaSeq			HiSeq X		
		GATK	SK2	SV	GATK	SK2	SV	GATK	SK2	SV	GATK	SK2	SV	GATK	SK2	SV
SNPs	True Positive	3033704	3018291	2996616	3034252	3021545	3012280	3042341	3041135	3012280	3035998	3029849	2935523	3043855	3041135	2935523
	False Positive	12742	28155	49830	12194	24901	34166	4105	5311	34166	10448	16597	110923	2591	5311	110923
	False Negative	34422	17550	29269	35160	17580	27573	39655	16399	27573	30697	16029	30061	29254	16399	30061
	Precision	0.995817	0.990758	0.983643	0.995997	0.991826	0.988785	0.998653	0.998257	0.988785	0.99657	0.994552	0.963589	0.99915	0.998257	0.963589
	Recall	0.988784	0.994223	0.990327	0.988548	0.99422	0.990929	0.987137	0.994641	0.990929	0.989993	0.994741	0.989863	0.990483	0.994641	0.989863
	F-score	0.992288	0.992488	0.986974	0.992259	0.993022	0.989856	0.992861	0.996445	0.989856	0.993271	0.994647	0.97655	0.994798	0.996445	0.97655
INDELs	True Positive	475745	480354	345161	477343	480726	356995	459351	486546	343256	457093	459567	344512	477406	486546	344512
	False Positive	23872	19263	153374	22274	18891	141540	40266	13071	155279	42524	40050	154023	22211	13071	154023
	False Negative	104479	64279	133516	105815	63750	136785	68723	60250	98057	97862	70151	95997	84827	60250	95997
	Precision	0.952219	0.961444	0.692351	0.955418	0.962189	0.716088	0.919406	0.973838	0.688529	0.914887	0.919839	0.691049	0.955544	0.973838	0.691049
	Recall	0.82562	0.885753	0.721073	0.82443	0.886689	0.722984	0.873733	0.893462	0.777806	0.828148	0.871175	0.782077	0.853896	0.893462	0.782077
	F-score	0.884412	0.922048	0.70642	0.885104	0.922898	0.71952	0.895988	0.93192	0.73045	0.869359	0.894846	0.73375	0.901865	0.93192	0.73375

We defined true positive (TP), true negative (TN), false positive (FP), and false negative (FN) variants as follows: (1)TP: variants called by a variant caller in high confident regions as the same genotype as the gold standard data; (2)TN: reference alleles in high confident regions other than gold standard variants; (3)FP: variants called by a variant caller in high confident regions but not as the same genotype as the gold standard data; (4)FN: gold standard variants in high confident that were not called by a variant caller; (5)Precision: TP/(TP+FP); (6)Recall: TP/(TP+FN); (7)F-score: 2* Precision*Recall/(Precision+Recall)

(5) Table.5 | Summary of running time of combinations in WES datasets.

Platforms	Coverage	GATK4		Strelka2	Samtools- Varscan	
		BQSR	Calling	Calling	Mpileup	Calling
BGISEQ500	30X	810m41.831s	1203m35.606s	43m22.730s	1367m5.072s	1771m3.787s
	24X	933m5.708s	1213m24.301s	43m52.252s	1357m52.898s	1754m51.019s
	18X	762m6.715s	1135m10.440s	37m51.731s	1136m27.422s	1651m14.34s
	12X	543m10.023s	967m18.077s	31m6.195s	1006m53.631s	1448m27.038s
	6X	318m24.029s	661m8.065s	26m36.095s	918m34.121s	709m38.311s
MGISEQ2000	30X	912m6.799s	1190m54.075s	42m54.705s	1408m10.665s	1800m43.289s
	24X	900m6.252s	1194m15.588s	48m7.604s	1384m42.017s	1783m38.604s
	18X	821m17.712s	1164m0.629s	43m22.191s	1270m39.725s	1751m24.142s
	12X	467m28.62s	1007m37.876s	32m51.366s	851m45.699s	1536m42.762s
	6X	344m29.776s	700m8.738s	25m20.767s	469m32.037s	920m25.056s
NovaSeq	30X	483m49.764s	1063m32.553s	30m52.464s	810m31.134s	1476m7.535s
	24X	396m13.403s	936m8.573s	29m21.287s	655m30.822s	1345m33.89s
	18X	322m52.541s	784m37.527s	25m50.803s	494m57.519s	1078m25.395s
	12X	244m19.439s	614m1.215s	23m35.691s	404m44.591s	586m42.762s
	6X	170m34.252s	423m40.743s	20m35.920s	402m33.086s	531m.048s
HiSeq4000	30X	806m49.429s	1237m39.473s	49m41.620s	1470m42.860s	1739m50.307s
	24X	807m32.985s	1246m4.586s	44m52.206s	1480m45.812s	1773m24.496s
	18X	825m1.4s	1244m33.119s	55m7.852s	1477m24.679s	1732m28.16s
	12X	632m30.839s	1135m22.265s	38m21.701s	1125m30.919s	1673m33.054s
	6X	370m33.769s	856m59.691s	27m21.182s	577m0.550s	1129m19.32s

	30X	831m35.247s	1259m32.016s	58m39.219s	1455m14.012s	1806m7.774s
	24X	835m25.743s	1261m11.415s	45m7.178s	1443m23.065s	1851m57.7s
HiSeq X	18X	674m27.979s	1164m42.926s	43m37.298s	1144m10.169s	1675m45.569s
	12X	500m59.992s	990m14.151s	31m6.316s	764m44.890s	1481m43.37s
	6X	285m48.362s	670m46.097s	25m20.870s	442m31.909s	834m35.577s

The variant calling efficiency of each combination run on a Tianhe-2 supercomputer with 24 virtual CPUs and 88 GiB of memory. All combinations were configured to schedule tasks over all 24 virtual CPUs. The coverages of the down-sampled datasets were approximately 20X, 40X, 60X, 80X, 100X, respectively. Among them, variants calling in SK2 sets 24 threads, GATK used the default setting thread in BQSR and 24 threads setting in variants calling, and SV used the default setting thread in mpileup and variants calling.
